# Environmental and Climatic Drivers of Microsporidial Keratoconjunctivitis in Athletes: Molecular Evidence from Outbreaks in Japan

**DOI:** 10.3390/microorganisms14030587

**Published:** 2026-03-05

**Authors:** Mohamed Talaat Mohamed, Masafumi Uematsu, Yasser Helmy Mohamed, Mao Kusano, Daisuke Inoue, Naoki Matsuya, Akio Oishi, Kenji Yagita

**Affiliations:** 1 Department of Ophthalmology and Visual Sciences, Graduate School of Biomedical Sciences, Nagasaki University, Nagasaki 852-8131, Japan; bb55323810@ms.nagasaki-u.ac.jp (M.T.M.);; 2 Matsuya Eye Clinic, Nagasaki 859-0401, Japan; 3 Department of Parasitology, National Institute of Infectious Diseases, Tokyo 162-0052, Japan

**Keywords:** microsporidia, keratoconjunctivitis, environmental reservoir, soil-borne pathogens, emerging infectious disease, molecular detection, ocular infection, climate association

## Abstract

*Vittaforma corneae* (*V. corneae*)-associated microsporidial keratoconjunctivitis (MKC) is increasingly recognized as an emerging infection affecting healthy individuals. However, the molecular links between environmental reservoirs and human diseases remain poorly understood. In this study, we examined the potential relationship between environmental factors and human MKC following 2 outbreaks in Nagasaki, Japan, involving 16 patients by integrating clinical, molecular, and environmental analyses. We collected ocular samples from affected patients and 16 soil and 11 water samples from related geographic areas. These samples were analyzed using nested PCR and DNA sequencing, targeting the *V. corneae* microsporidian ribosomal genes. Our molecular comparisons revealed a high degree of sequence similarity between clinical and environmental soil isolates, suggesting a potential soil-associated reservoir for the infection. Outbreaks of MKC occurred following periods characterized by high temperatures, high humidity, and increased rainfall. While we cannot definitively establish causality, these findings support the hypothesis that environmental exposure may contribute to microsporidial ocular infections. Temporary visual impairment among affected athletes disrupted their training and raised concerns among sports teams regarding environmentally driven ocular infections. Overall, our findings reveal a clear molecular and ecological pathway for MKC transmission in a non-endemic country. They underscore the importance of environmental surveillance in sports fields and other high-contact environments.

## 1. Introduction

Microsporidial keratoconjunctivitis (MKC) is an increasingly recognised ocular infection affecting immunocompetent individuals in Asia, particularly those exposed to soil, mud, or contaminated water during outdoor activities or sports [1,2,3]. Among the microsporidia causing ocular disease, *Vittaforma corneae* (*V. corneae*) is the most frequently implicated species and has been reported in outbreaks and sporadic cases in India, Singapore, Thailand, and other tropical regions [4,5,6,7]. MKC typically presents with acute unilateral or bilateral conjunctival hyperemia, foreign-body sensation, photophobia, and superficial punctate keratitis, but it is often misdiagnosed as viral conjunctivitis because of overlapping clinical features and limited clinician awareness [1]. Despite increasing recognition of this pathogen, the environmental transmission pathways of *V. corneae* remain poorly defined, and molecular evidence directly linking soil or other environmental reservoirs to human infection remains scarce [5,8].

Several reports indicate that MKC incidence increases during periods of high humidity, elevated temperatures, and heavy rainfall, which are the same environmental conditions that favour microsporidial persistence in the soil [9,10].

Japan experienced its first MKC outbreak in Nagasaki in 2022 [11]. Although Japan has not historically been considered endemic to MKC, recent climatic variability, including warmer, more humid periods and episodic soil saturation, may facilitate the emergence of MKC in temperate regions. Athletes training on playground fields face an increased risk of exposure after rainfall. However, to date, no study from Japan has provided molecular confirmation linking local environmental reservoirs to clinically confirmed MKC cases.

This study investigated clusters of MKC among immunocompetent football players in Nagasaki, Japan, during 2022 and 2023, with the goal of clarifying how environmental exposure contributes to disease emergence in a non-endemic region. We sought to confirm *V. corneae* as the causative agent among affected athletes, examine the molecular relationship between clinical and environmental isolates, and evaluate how temperature, humidity, and rainfall patterns aligned with the outbreak timing. Together, these aims were integrated into a methodological framework to link clinical diagnoses to environmental reservoir detection and to provide molecular evidence of transmission pathways during MKC outbreaks.

## 2. Materials and Methods

Using a nested PCR assay targeting the SSU rRNA gene, we performed molecular detection of *V. corneae* in corneal samples, eye discharge, and environmental soil and water samples, followed by BLAST (http://blast.ncbi.nlm.nih.gov/Blast.cgi, accessed on 2 February 2026) and phylogenetic analyses to determine the relationships between clinical and environmental strains. We also analysed local climatic variables, including temperature, humidity, and rainfall, to assess temporal associations with outbreak onset and to compare these associations with those reported in previous studies in India.

**Figure 1:**
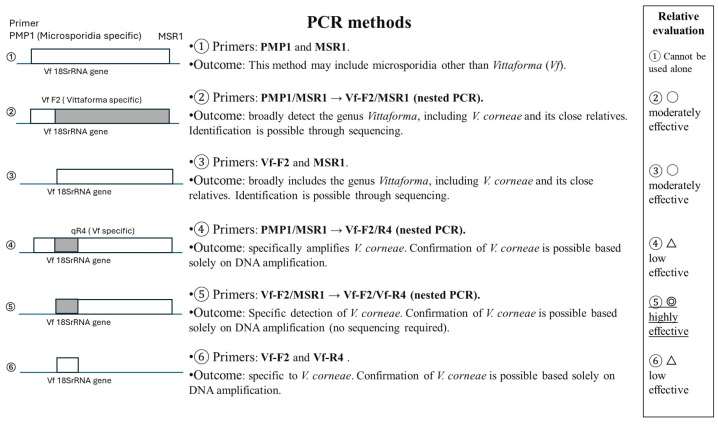
Schematic representation and comparative evaluation of six PCR strategies for the detection of microsporidia and *V. corneae* in environmental samples (soil and water). ① Conventional PCR using PMP1/MSR1, which broadly detects microsporidia but cannot distinguish *Vittaforma* species without sequencing. ② Nested PCR using PMP1/MSR1 followed by Vf-F2/MSR1, enabling genus-level detection of *Vittaforma*, including *V. corneae*, with species identification requiring sequencing. ③ Single-round PCR using Vf-F2/MSR1 for genus-level detection of *Vittaforma*. ④ Nested PCR using PMP1/MSR1 followed by Vf-F2/Vf-R4 (Vf-specific), specifically amplifying *V. corneae*. ⑤ Nested PCR using Vf-F2/MSR1 followed by Vf-F2/Vf-R4, providing highly specific detection of *V. corneae* without the need for sequencing. ⑥ Single-round PCR using Vf-F2/Vf-R4 for direct detection of *V. corneae*. The right panel summarizes the relative diagnostic performance of each approach. PMP1 and MSR1, microsporidia-specific primers; Vf-F2 and Vf-R4, *V. corneae*-specific primers; 18S rRNA, small subunit ribosomal RNA gene.

➢ Patient data collection: In 2022 and 2023, we documented MKC outbreaks in Nagasaki that affected 16 healthy football players from two teams. Data were collected from Nagasaki University Hospital and Matsuya Eye Clinic. The first outbreak involved five patients, all from the same football team. The second outbreak involved 11 patients, including 10 from the same football team and one from a high school. Corneal epithelial scrapings and eye discharge samples were collected from the patients and subjected to PCR analysis. PCR and DNA sequencing of corneal samples from the first outbreak have been described in our previous study [11].
➢ Environmental Sampling: We collected 15 soil and 11 water samples from the football team club, high school, and their surroundings after the 2023 outbreak, and one sample from the football team club practice field after the 2022 outbreak. Samples from high schools were collected after the soil on the football playground was replaced, whereas the surrounding area remained unchanged. The football team’s practice field featured well-cultivated grass that was fertilised and irrigated. The high school playground had sandy soil. Samples were collected from the football practice field, nearby parks, fertilisers, animal droppings, and various water sources, including irrigation, rainwater from asphalt, and tap water (Table 1).
➢ PCR protocol: We employed multiple PCR methods to ensure the accurate detection of the causative organism (Figure 1).

Sample preparation from soil and water: For soil samples, approximately 1 mL of soil was placed into a 15 mL centrifuge tube, and 10 mL of washing solution (distilled water containing 0.05% Tween-80) was added. The tube was vortexed for 15 s and centrifuged for 1 min at 300× *g*. A total of 2 mL of the supernatant was transferred to a 2.0 mL microcentrifuge tube and centrifuged for 3 min at 10,000× *g*. The supernatant was carefully removed, and 100 μL of TE (Tris-EDTA) buffer with 5 mM EDTA was added to the sediment.

For water samples, 10 mL of water and 50 μL of 10% Tween-80 were gently mixed (0.05% final concentration of Tween-80), and 2 mL aliquots of the mixture were centrifuged in a 2 mL tube at 10,000× *g* for 3 min. All the sediment recovered from the 10 mL sample was resuspended in 100 μL of TE buffer with 5 mM EDTA.

Bead beating and DNA extraction: Concentrated samples from soil or water were transferred to a 2 mL screw-capped tube containing 100 μL of 0.5 mm zirconia beads. Bead beating homogenisation was performed using a Disruptor Genie (Scientific Industries, Inc., Bohemia, NY, USA). The samples were homogenised for 5 min at room temperature. DNA extraction was performed using the QIAamp DNA Mini Kit (QIAGEN, Venlo, The Netherlands) with minor modifications. After sample homogenisation, 200 μL of buffer ATL, 15 μL of proteinase-K, and 200 μL of buffer AL were added to the lysate in the screw-capped tube and vortexed for 15 s. The tube was incubated at 70 °C for 1 h. After centrifugation at 10,000× *g* for 1 min, the lysate was transferred to a fresh tube and mixed with 200 μL of 99.5% ethanol. Subsequent DNA extraction was performed according to the manufacturer’s instructions. Finally, the extracted DNA was eluted in 100 μL of AE buffer.

Semi-nested PCR and molecular identification: A semi-nested PCR procedure was developed to amplify the *V. corneae*-specific region in the small subunit RNA gene. The first PCR primer set comprised Vf2:5′-CCATGCATGTTTCCTCAATCAG (forward, newly designed) and msprv1: 5′-GTTGAGTCAAATTAAGCCGCACA (reverse, 3′-end-modified), used to amplify a partial region of approximately 750 bp of the small subunit ribosomal RNA gene, which was applicable for sequence analysis. One of the second PCR primers, R4: 5′-CCTGCGTCTTATTCTGCCT (reverse), was designed to amplify 115 bp DNA using Vf2. HotStarTaq DNA Polymerase (QIAGEN) was used for high-specificity PCR. Our semi-nested PCR protocol was as follows: in the first PCR, initial denaturation (94 °C for 15 min) to activate the enzyme was followed by 40 cycles of denaturation (94 °C for 30 s), annealing (57 °C for 1 min), extension (72 °C for 30 s), and final elongation (72 °C for 5 min) as the last step. In the second PCR, the protocol was largely the same, except for higher annealing stringency (63 °C for 30 s) and 25 cycles to complete the round. Vf2/MSRv amplicons from the first PCR were purified, and their sequences were determined using the Sanger sequencing method. The sequence similarity of the amplicon DNA was determined using BLAST in the NCBI database.

We compared the PCR results of soil and water with those of the patient’s corneal scrapings and eye discharge samples collected during outbreaks.

➢ Weather data analysis: Weather data from the Japan Meteorological Agency and World Weather Online (https://www.worldweatheronline.com/) were analysed during MKC outbreaks. These data were then compared with other studies conducted in India that reported MKC cases in Bhubaneswar, Odisha, from March 2007 to October 2010, and in the district of Hyderabad from January 2016 to December 2019 [4,10].

## 3. Results

**Figure 2:**
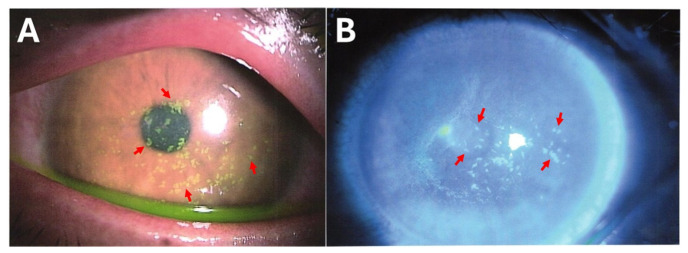
Clinical presentation of the MKC case. (**A**) Slit-lamp photograph showing numerous coarse punctate epithelial lesions (red arrows) involving the central cornea. The lesions stain intensely with fluorescein and appear as discrete yellow-green spots. Mild conjunctival injection was observed. (**B**) Blue light illumination demonstrates diffuse punctate epithelial infiltrates (red arrows) and overlying epithelial haze. The lesions appeared as multiple irregular hyperfluorescent spots on the corneal surface.

**Figure 3:**
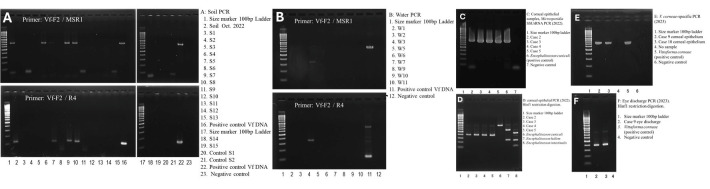
PCR detection of microsporidian DNA in environmental samples and clinical specimens. (**A**) Nested PCR of soil samples using Microsporidia *V. corneae*-specific primers (Vf-F2/MSR1 → Vf-F2/R4) produced amplicons of the expected size in October 2022 soil and samples S4, S5, S7, and S8. (**B**) The same nested PCR applied to water samples yielded no detection of *V. corneae* DNA. (**C**) PCR of corneal epithelial scrapings from the first outbreak showed positive bands in all four cases. (**D**) Analysis of the corneal epithelial PCR products revealed banding patterns that differed from those of the reference controls. This discrepancy prompted further analysis; subsequent *V. corneae*-specific PCR and BLAST confirmed *V. corneae* as the causative organism. (**E**) *V. corneae*-specific PCR of corneal scrapings from Cases 9 and 10 during the second outbreak produced the expected diagnostic band. (**F**) *V. corneae*-specific PCR of eye discharge from Case 9 was also positive. S = soil; W = water; Vf-F2/R4 = *V. corneae*-specific primers; MSR1 = Microsporidia-specific reverse primer.

Clinical findings: All affected individuals were immunocompetent males aged 17–36 years old. One patient experienced reinfection thrice. Patients experienced a decrease in best-corrected visual acuity (BCVA), redness, eye discharge, pain, itching, and a foreign-body sensation. More severe manifestations include corneal opacities, multiple subepithelial corneal infiltrations, and dazzling light, which make it difficult to see the ball while playing football (Figure 2). Detailed clinical presentations are provided (Table S2). PCR analysis of corneal and eye discharge samples from the patient was positive for *V. corneae* (Figure 3).

All patients recovered after the treatment. Patients were treated with voriconazole eye drops, 1.5% levofloxacin eye drops, pimaricin eye ointment, and iodine/polyvinyl alcohol eye drops. In addition, 0.1% fluorometholone eye drops were prescribed only for patients with severe corneal opacities, and these cases were carefully monitored for any worsening of the infection. They were advised to wash their eyes regularly and use iodine eye drops after the training.

**Figure 4:**
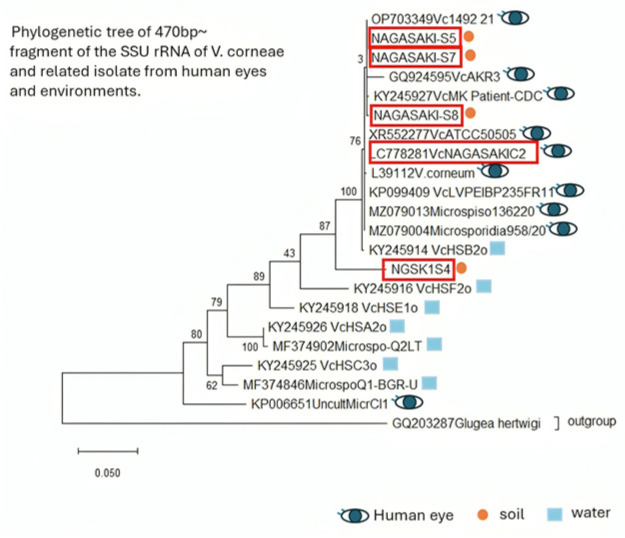
The phylogenetic tree includes *V. corneae* isolates from human eyes (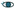), soil (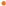), and water (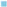), illustrating their environmental and clinical origins. Five isolates obtained in this study (NAGASAKI-S5, -S7, -S8, C778281VcNAGASAKIC2, and NGSK1S4) are highlighted in red boxes. *Glugea hertwigi* (GQ203287) was used as the outgroup. The clustering of Nagasaki isolates with other human-derived strains suggests a close genetic relationship, supporting the hypothesis that environmental sources may act as reservoirs for pathogenic *V. corneae*.

Molecular and environmental Analysis: PCR tests for soil and water samples showed that method ⑤ was the most effective (Figure 1 and Figure 4). Method ⑤ provides high sensitivity for detecting *V. corneae* even when spore numbers are low, and the specific primer sets minimize cross-amplification from other microeukaryotes. Method ⑤ was nested PCR, which required first-round amplification using Vf-F2/MSR1 primers before applying the second round using Vf-F2/R4 primers.

Four of sixteen soil samples’ PCR tests were positive for *V. corneae* in the football team practice field, high school sub-ground soil, and planting area around the high school playground (Figure 3, Table 1, and Table S1). All 11 water samples were negative for *V. corneae* but positive for other microsporidia species (Figure 3, Table 1, and Table S1). Of 16 patients, 8 underwent corneal scraping and PCR testing, and all were positive for *V. corneae*. Of the 4 patients who experienced eye discharge, 1 patient’s eye discharge sample was PCR-tested and was positive for *V. corneae*.

The GenBank registration sequence was done for the SSU rRNA Gene Sequences of *V. corneae* Isolates from Nagasaki (S4, S5, S7, and S8) with accession numbers PV875095, PV875116, PV875206, and PV875207, respectively.

The results of the sequence similarity of the amplicon DNA by BLAST in the NCBI database are detailed in (Table 2).

Phylogenetic analysis demonstrated that all soil-derived isolates clustered tightly with the previously reported human-derived *V. corneae* strain NAGASAKI-C2 (LC778281), indicating a shared recent ancestry between the environmental and clinical isolates. The high sequence identity observed in samples S5, S7, and S8 (99%) suggests that these environmental strains are either identical or nearly identical to the pathogenic strain infecting athletes during the outbreaks. Although S4 showed a slightly lower similarity (94%), its placement within the same clade indicates that it represents a closely related lineage rather than a distinct species (Figure 4).

**Figure 5:**
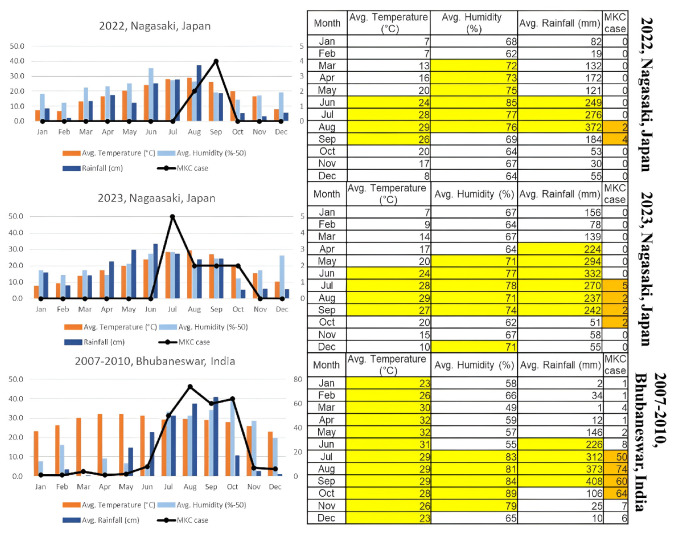
Charts (top, middle and bottom) illustrate the relationship between MKC prevalence and weather conditions in Nagasaki, Japan, and Odisha, India. Monthly climate variables—average temperature (°C), relative humidity (%), and rainfall (mm). MKC cases tend to increase mainly after periods with temperature ≥23 °C, humidity ≥70%, and rainfall ≥200 mm. Yellow bars indicate climate variables that exceed the predefined climate thresholds. Orange bars represent months with MKC outbreaks.

Weather Correlation: Five cases of MKC were reported in August and September 2022, and 11 cases were reported from July to October 2023 in Nagasaki. MKC tends to occur after a month with or exceeding the following weather conditions: a temperature of 23 °C, a humidity of 70%, and a rainfall of 200 mm. This pattern is consistent with similar findings in India (Figure 5) [4,10]. A lag time of approximately 3–5 weeks was observed between the previously mentioned weather conditions and case onset.

## 4. Discussion

To our knowledge, this study is the first to clearly reveal the relationship between soil and MKC caused by *V. corneae* in Japan using molecular epidemiological techniques. Molecular confirmation from both environmental and clinical samples, combined with BLAST and phylogenetic clustering, was consistent, with soil as the principal transmission source, while water was free of *V. corneae*. MKC outbreaks increase during the rainy season after a month of high temperatures, humidity, and heavy rainfall. The outbreaks occurred among immunocompetent football players, emphasising that MKC is no longer confined to immunocompromised populations. These findings underscore the importance of considering environmental exposure during the clinical evaluation of keratoconjunctivitis cases.

Clinical impact: Although MKC is self-limiting in most cases, it can cause temporary visual impairment and disrupt athletic training and performance as well as outdoor occupational activities. KC involves inflammation of both the conjunctiva and cornea, and environmental exposure can activate innate immune pathways on the ocular surface. When epithelial cells detect pathogens, membrane receptors like Toll-like and cytokine receptors trigger signaling cascades. The MAPK pathway is crucial for amplifying inflammation by activating transcription factors such as AP-1, thereby enhancing cytokine production, including IL-1 and TNF-α, and contributing to the inflammatory response [12]. In addition, pathogen-related stimulation can activate the PI3K/Akt pathway, which interacts with mTOR to regulate immune cell activity and host defense mechanisms during infection [13].

Outbreaks among immunocompetent individuals in non-endemic countries, such as Japan, raise significant concerns, particularly in athletic communities. The diagnostic approach presented here enables clinicians to differentiate MKC from other infectious keratoconjunctivitis. Our findings support integrating environmental surveillance into routine outbreak management for MKC. Soil testing using the molecular workflow described here is feasible, rapid, and adaptable for school athletic fields, sports facilities, and community recreation areas.

Diagnostic Challenges and PCR Optimisation: Early and accurate diagnosis of MKC remains challenging because of nonspecific symptoms and the resilience of microsporidial spores. We optimized a nested PCR system (Vf-F2/MSR1 → Vf-F2/R4) (method ⑤) with superior specificity and sensitivity for the detection of *V. corneae* in environmental samples.

Molecular and environmental: MKC infection is more likely due to soil or mud, as PCR has detected *V. corneae* in soil samples, consistent with PCR results from patients’ corneal scrapings and eye discharge samples. This was supported by phylogenetic tree and BLAST analyses, which showed high similarity between Nagasaki-C2 (LC778281) and soil samples S5, S7, and S8 (PV875116, PV875206, and PV875207), respectively.

However, the water samples were negative for *V. corneae* but positive for other microsporidia species. The water results differ from those previously reported in Thailand, which showed *V. corneae* in the water samples [14]. This may be attributed to variations in PCR techniques, differences in water treatment in Japan and Thailand, or differences in the transmission routes of *V. corneae* strains in Thailand.

BLAST analysis and phylogenetic topology support a direct environmental–clinical link. They provide compelling molecular evidence that the environmental isolates responsible for the 2022–2023 outbreaks were not incidental soil microsporidia but belonged to the same pathogenic lineage as that circulating among the affected athletes.

In particular, isolate S4 warrants attention because of its genetic proximity to human ocular strains. Its visual clustering with the known eye-infecting *V. corneae* suggests that it may be a potentially pathogenic organism present in the soil. This finding supports the idea that the early detection of such strains could play a key role in preventing future outbreaks.

Climatic Factors and Seasonal Risk: Weather analysis reports have highlighted a relationship between weather conditions and ocular microsporidial infections, with the rainy season identified as a significant risk factor [1,6,9,15]. Outbreaks coincided with climatic conditions characterised by elevated temperatures, humidity, and increased rainfall. These thresholds likely promote *V. corneae* persistence and sporulation in the soil. As global warming intensifies rainfall variability, temperature, and humidity, similar outbreaks may emerge in new geographical areas.

Preventive strategies should include environmental monitoring of sports fields, public education on eye hygiene, and early use of antiseptic eye drops after soil exposure. Establishing seasonal alert systems for MKC could help prevent future outbreaks of this disease. Regular monitoring after periods of heavy rainfall, high temperatures, or high humidity can provide an early warning of increased pathogen load in the soil, enabling preventive interventions. By linking molecular detection, climatic drivers, and clinical outcomes, this study provides a translational framework for managing environmentally mediated ocular infections.

Study Limitations: This study was conducted in a specific geographic area and had a limited number of cases. Also, PCR contamination risk, sampling timing representativeness, and the descriptive nature of climate thresholds. Broader surveillance across different climatic zones and soil types is required to validate the environmental thresholds identified in this study.

Future Research Directions: Our subsequent study aims to address epidemiological questions regarding the observed increase in cases associated with specific weather conditions. We aim to understand the reasons behind the few weeks’ lag between these conditions and the onset of outbreaks. Given that the likelihood of *V. corneae* being present in rainwater or daily water supply is almost zero, it is reasonable to assume that the source is soil. We hypothesize that a natural host of *V. corneae* has not yet been identified or reported and may reside in the soil. We believe that increases in temperature, humidity, and rainfall contribute to higher host population densities, leading to a higher concentration of *V. corneae* in the environment. Further investigations are necessary to elucidate the nature and life cycle of *V. corneae,* thereby informing the development of more effective methods for preventing *V. corneae* infections.

## 5. Conclusions

This study reports the first documented MKC outbreaks in Japan and provides molecular evidence consistent with *V. corneae*-contaminated soil functions as an environmental reservoir among healthy individuals, while water sources remain free of the organism. The close genetic relatedness between soil and patient isolates supports direct environmental transmission. Although causality cannot be definitively established, the findings emphasize the potential influence of environmental exposure and climatic conditions on disease emergence. The nested PCR assay developed in this study enables sensitive detection of *V. corneae* in environmental samples, offering a practical tool for targeted surveillance and early intervention. As climate patterns continue to shift, similar outbreaks may emerge in regions previously considered non-endemic. Integrating molecular diagnostics with environmental monitoring is essential for protecting at-risk populations and ensuring safe conditions for outdoor activities.

## Figures and Tables

**Table 1:** Soil and water PCR results, Nagasaki, Japan, 2022–2023.

Category	Samples Tested	Vf-F2/MSR1 (+)	Vf-F2/R4 (+)	Notes
Controls	2	0	0	Unused pet sand, aquarium soil
School Soil	5	2	1	(+) in planting/sub-ground
Football Club Soil	10	3	3	All (+) from the practice field
Fertilizers	2	0	0	All (−), Amino Midori, Enzaamine
Animal Droppings	4	0	0	All (−)
Water	11	0	0 *	All (−) for *V. corneae*

This table summarizes the PCR results for the soil and water samples. The soil samples tested positive for microsporidia, including *V. corneae*. Five water samples tested positive for Microsporidia, but none tested positive for *V. corneae*. Vf-F2: *V. Corneae*-specific primer. MSR1 and PMP1: microsporidia-specific primers. (−): negative and (+): positive. More detailed data are provided in (Table S1). * 5 of 11 samples were (+) for PMP1/MSR1 (microsporidia species other than *V. corneae*).

**Table 2:** BLAST analysis of *Vittaforma corneae* isolates from soil samples from different locations.

Sample	Location	Source	BLAST Hit (ACC. No.)	Maximum Identity’ (%)	Reference Source
S 4 (PV875095)	High school	Soil in the plantings beside the ground	*Vittaforma corneae* NAGASAKI-C2 (LC778281)	670/715 (94%)	Human cornea
S 5 (PV875116)	High school	Sub-ground soil	*Vittaforma corneae* NAGASAKI-C2 (LC778281)	711/712 (99%)	Human cornea
S 7 (PV875206)	The football team’s playground	Soil on the practice field	*Vittaforma corneae* NAGASAKI-C2 (LC778281)	711/712 (99%)	Human cornea
S 8 (PV875207)	The football team’s playground	Soil on the practice field	*Vittaforma corneae* NAGASAKI-C2 (LC778281)	710/712 (99%)	Human cornea

This table: BLAST results of environmental strains from a field study conducted after MKC outbreaks in Nagasaki, Japan. The top BLAST Hit indicates the closest reference species in the NCBI BLAST database (https://blast.ncbi.nlm.nih.gov/Blast.cgi, accessed on 2 February 2026) to the DNA sample. Maximum Identity (%) indicates the percentage identity between the sample and reference sequences. The Reference Source suggests the origin of the reference sequence used for comparison. S = Soil.

## Data Availability

The nucleotide sequences of *the Vittaforma corneae* isolates obtained in this study were deposited in the GenBank database under accession numbers PV875095, PV875116, PV875206, and PV875207. The original contributions presented in the study are included in the article, further inquiries can be directed to the corresponding author.

## Supplementary Materials

The following supporting information can be downloaded at: https://www.mdpi.com/article/10.3390/microorganisms14030587/s1, Table S1: Soil and Water PCR results; Table S2: Clinical presentation and PCR results of the MKC patients.

## Abbreviations

The following abbreviations are used in this manuscript:

MKC	Microsporidial keratoconjunctivitis
*V. corneae*	*Vittaforma corneae*
KC	Keratoconjunctivitis
BCVA	Best corrected visual acuity
